# Malaria vaccines for eradication

**DOI:** 10.1186/1475-2875-11-S1-O47

**Published:** 2012-10-15

**Authors:** Pedro Alonso

**Affiliations:** 1 Barcelona Centre for International Health Research (CRESIB), Spain

## 

In achieving the goal of malaria eradication, vaccines could play a crucial role. Current attempts to develop malaria vaccines are primarily focused on *Plamosdium falciparum* and are directed towards reducing morbidity and mortality. This is the case of the RTS,S vaccine candidate, the most developed at the moment and currently undergoing the final analysis of a multicentric, international Phase III clinical trial. Continued support for these efforts is essential, but if malaria vaccines are to be used as part of a repertoire of tools for malaria eradication, they will need to have an impact on malaria transmission, contributing to lower the reproductive rate to less than one.

The consultative group on vaccines of the Malaria Eradication Research Agenda (malERA) initiative identified key features of vaccines especially suited for malaria eradication. The group introduced the concept of “Vaccines that Interrupt Malaria Transmission” (VIMTs), broadening the classical definition of Transmission Blocking Vaccines (TBVs) -that target sexual and mosquito stages of the parasite-, in order to also include any other parasite life stages that may interrupt transmission of malaria. This would include pre-erythrocytic and asexual stage vaccines to reduce prevalence and densities of sexual forms of the parasite, as well as vaccines against mosquito gut antigens impeding parasite development in the vector.

Moreover, malaria eradication would necessarily need to target not only *P. falciparum* but also *P. vivax*, for which therapeutic vaccines against hypnozoites or preventive vaccines with effect against multiple parasite stages could have enormous impact.

Development of VIMTs require the advancement of basic understanding of interactions of the malaria parasite with humans and vectors, as well as the development of tools to measuring infectivity at the individual level and assessing transmission within a given population. VIMTs may have primarily an effect at the population level and could be crucial not only for eradication, but also in areas where malaria control is still the main issue to be addressed. In order to accelerate the actual implementation of such vaccines, it is also important to address regulatory issues and to develop appropriate delivery platforms.

